# A survey of energy drink and alcohol mixed with energy drink consumption

**DOI:** 10.1186/s13584-015-0052-5

**Published:** 2015-12-01

**Authors:** Racheli Magnezi, Lisa Carroll Bergman, Haya Grinvald-Fogel, Herman Avner Cohen

**Affiliations:** Public Health and Health Systems Management Program, Bar Ilan University, Ramat Gan, Israel; Pediatric Ambulatory Community Clinic, Clalit Health Services, Petach Tikva, Israel; Sackler Faculty of Medicine, Tel Aviv University, Tel Aviv, Israel

**Keywords:** Energy drinks, Alcohol mixed with energy drinks, Substance abuse, Youth

## Abstract

**Background:**

Energy drink consumption among youth is increasing despite recommendations by the American Academy of Pediatrics to eliminate consumption by youth. This study provides information on consumption of energy drinks and alcohol mixed with energy drinks (AmED) in a sample of Israeli youth and how consumer knowledge about the risks affects consumption rates.

**Methods:**

The study was conducted in three Tel Aviv public schools, with a total enrollment of 1,253 students in grades 8 through 12. Among them, 802 students completed a 49-item questionnaire about energy drink and AmED consumption, for a 64 % response rate Non-responders included 451 students who were absent or refused to participate. All students in the same school were administered the questionnaire on the same day.

**Results:**

Energy drinks are popular among youth (84.2 % have ever drunk). More tenth through twelfth grade students consumed energy drinks than eighth and ninth grade students. Students who began drinking in elementary school (36.8 %) are at elevated risk for current energy drink (*P* < .001) and AmED (*P* = .002) use. Knowledge about amounts consumed and recommended allowances is associated with less consumption (OR 1.925; 95 %CI 1.18–3.14).

**Discussion:**

The association between current AmED consumption and drinking ED at a young age is important. Boys and those who start drinking early have a greater risk of both ED and AmED consumption. The characteristics of early drinkers can help increase awareness of potential at-risk youth, such as junior and senior high school students with less educated or single parents.

**Conclusions:**

Risks posed by early use on later energy drink and AmED consumption are concerning. We suggest that parents should limit accessibility. Increased knowledge about acceptable and actual amounts of caffeine in a single product might decrease consumption.

## Background

Children and teens are consuming increasing quantities of energy drinks (ED) internationally [1]. These drinks represent 8.8 % of all sugar-sweetened beverages consumed by high school students [2]. Another study found that 31 % of 12- to 17-year-olds regularly consume ED, compared to 22 % of those 25- to 35-years-old [3]. The high level of caffeine in these drinks (70 to 80 mg per 8-oz serving and much higher in some) is especially risky for children and adolescents [4, 5].

Much of the information about the effects and risks of ED is derived from studies on adults [3, 4, 6]. Research regarding children and adolescents is lacking, even though the potential effects of caffeine on their developing neurological and cardiovascular systems and the risk of physical dependence and addiction are concerning. The American Academy of Pediatrics Committee on Nutrition and the Council on Sports Medicine and Fitness have concluded, “Caffeine and other stimulant substances contained in ED have no place in the diet of children and adolescents” [7].

ED consumption is also associated with alcohol use, as found in a recent study of 3,342 youth ages 15 to 23 [8]. Combining alcohol and caffeine increases alcohol tolerance [9, 10]. The mixture causes drinkers to be less aware of how drunk they are, making them more likely to drink more and engage in risky behaviors [11], a condition called “wide awake drunk.” In addition, alcohol mixed with energy drinks (AmED) consumption is associated with alcohol dependence, binge drinking and increased high-risk sexual behaviors [12, 13]. About 51 % of college age students consume AmED [14]. Although consumption of AmED by youth younger than college age is particularly risky, the literature examining the incidence and risks is sparse [15–17].

The present report investigated the pattern of ED and AmED consumption in a sample of Israeli youth, including sociodemographic factors, the association of grade at first use of ED with current ED and AmED consumption, and the effect of consumer knowledge about associated risks.

## Methods

Data were collected from students in three large public schools in the greater Tel Aviv area during December 2012 and January 2013. A total of 1,253 students were enrolled in grades 8 through 12. Grades 8–9 (Junior High School) included students 14 to 15 years-of-age and grades 10–12 (Senior High School) included students 16 to 18 years-of-age. The response rate was 64 %. All students in the same school were administered the questionnaire on the same date. Non-responders include 451 students who were absent that day or who refused to participate.

The students were administered a 3-part, 49-question survey. The first section included 25 questions about ED consumption. Energy drinks in this study refer to high-caffeine products marketed and labeled as such. The second part comprised 11 questions about AmED and the third part included 14 sociodemographic questions. The questionnaire was compiled based on a literature review of ED use in youth and is modeled after a study by Attila and Cakir [18]. Questions included whether the participant had ever ingested an ED, age and grade at first use, and amount consumed on a regular basis. Information concerning knowledge about ED use, including potential risks, recommended limits of caffeine and formal education about the topic was also gathered. Similar questions were asked regarding AmED use. The items on the questionnaire did not lend themselves to validity testing. One of the authors, a pediatrician, changed the language and questions to be more appropriate to the sampled age group. The questionnaire was translated into Hebrew. It was back-translated into English by a different translator to ensure that the meaning of the questions remained the same. The study was approved by the Israel Ministry of Education Review Board. Parental consent was not required.

Study socio-demographic variables were gender, year and country of birth, family structure (defined as married or other), and parental level of education (low parental education was defined as both parents with at most a high school education).

Regarding energy drink use, the respondents were asked if they ever drank energy drinks, and if so, how many cans per day. They were also asked their age and if they were in elementary, junior high or high school at first consumption. Further questions regarding energy drinks consumption included the location at first consumption and the location of regular consumption (home, at a sports activity, bar, party, or coffee shop). Respondents who drank energy drinks were asked if they ever needed medical attention due to side effects such as confusion, stomach aches, headache epilepsy and dizziness, and if so, who treated them.

The respondents were asked about reasons for consuming energy drinks—taste, energy boost, practice enhancement, mixing with alcohol, curiosity and consumption of more alcohol. Knowledge about the main ingredients of energy drinks, amount of caffeine and negative health effects, such as arrhythmia, liver and kidney damage, respiratory disease and blood pressure was tested using a series of true and false questions.

The respondents were asked how often they mix energy drinks with alcohol and the reasons for doing so—better taste, less alcohol side effects, social reasons, curiosity, to consume more alcohol or to feel alert. Finally, respondents were asked if they were ever formally taught about energy drinks and the dangers in consuming them with or without alcohol, and queried if they would want to receive further information regarding this matter.

### Statistical analysis

All statistical analyses were performed using SPSS statistical software, version 20.0. Chi-square tests for independence were carried out to examine the dependency between the characteristics of the respondents and their ED consumption. A p-value < 0.05 was considered significant.

Current consumption of ED and AmED were regressed separately on the study variables using binary logistic regression. Specifically, we examined gender, family structure, parental education, current age and country of birth as potential predictors.

The regression models (or analyses) were then stratified by school level at first consumption. Odds ratios with 95 % confidence intervals were calculated for each independent variable.

## Results

### Energy drink use

This study included 802 junior high and high school students, 50.7 % was male. Among the 802 participants, 84.2 % (*n* = 675) had consumed an ED. Just over a third (36.8 %) first tried ED in elementary school, 51.3 % in junior high, and 11.8 % in high school. The mean age at first use was 12.5 (standard deviation, 2.2) years. At the time of the survey, 30 % of the 802 respondents were drinking a can or less per day, 2.2 % drank two cans and 1.5 % drank more than two cans per day. Among participants who reported drinking, 39.1 % reported that the first time they had drunk was at home and another 31.1 % first started drinking at a party. Currently, 43.9 % reported that they usually drank at parties. A total of 4.2 % reported that they required medical attention after consuming ED.

### Sex differences

Ingestion behaviors differed significantly between the sexes (Table 1). Among boys, 90.1 % had ever ingested ED compared to 78.4 % of girls. Proportionately more boys (41.5 %) than girls (26.3 %) drank daily (*χ*^2^(1) = 20.163, *p* < 0.001).

**Table 1:** Characteristics of students who reported energy drink consumption

Characteristic	Total	Ever consumed energy drinks	Consumes at least one can daily^a^	Ever mixed energy drinks with alcohol^a^
Total, N (%)	802	675 (84.2)	264 (33.8)	309 (38.5)
Gender, N (%)				
Male	395 (49.3)	356 (90.1)	160 (41.5)	190 (48.8)
Female	407 (50.7)	319 (78.4)	104 (26.3)	119 (29.5)
*χ* ^2^		20.76^***^	20.16^***^	31.32^***^
Current school level, N (%)				
Junior high (14-15-years-old/grades 8-9)	372 (47.1)	300 (80.6)	141 (39.0)	110 (30.1)
High school (16-18-years-old/grades 10-12)	418 (52.9)	364 (87.1)	120 (29.3)	196 (47.0)
*χ* ^2^		6.08^*^	8.05^**^	23.24^***^
Country of birth, N (%)				
Israel	733 (93.4)	620 (84.6)	233 (32.7)	279 (38.5)
Other	52 (6.6)	46 (88.5)	25 (48.1)	22 (43.1)
*χ* ^2^		0.57	5.14^*^	0.44
Family structure, N (%)				
Parents married	613 (79.2)	514 (83.8)	188 (31.4)	233 (38.3)
Other	161 (20.8)	140 (87.0)	66 (41.8)	69 (43.4)
*χ* ^2^		0.94	6.05^*^	1.39
Parental education level, N (%)				
Low (High school, both parents)	226 (32.4)	198 (87.6)	77 (34.8)	102 (45.3)
High (Academic, at least one parent)	472 (67.6)	399 (84.5)	140 (30.4)	178 (37.8)
*χ* ^2^		1.17	1.34	3.60
School level at first consumption, N (%)				
Elementary	246 (36.8)	242 (98.4)	132 (54.5)	126 (52.3)
Junior high	343 (51.3)	341 (99.4)	113 (34.0)	134 (39.6)
High school	79 (11.8)	79 (100.0)	16 (20.8)	39 (51.3)
* χ* ^2^		2.56	38.08^***^	10.17^**^

^*^ *p* < 0.05; ^**^ *p* < 0.01; ^***^ *p* < 0.001; ^a^ *N* = 782

### Additional sociodemographic factors

Other significant sociodemographic differences included current grade, country of birth and family structure (Table 1). Proportionately more senior than junior high school students had consumed ED (*χ*^2^(1) = 6.082, *p* < 0.05) or AmED (*χ*^2^(1) = 23.243, *p* < 0.001), but proportionately more junior than senior high school students consumed at least one can per day (*χ*^2^(1) = 8.053, *p* < 0.01). Immigrant students were significantly more likely to consume more than one can per day (*χ*^2^(1) = 5.141, *p* < 0.05) as were students from single parent homes (*χ*^2^(1) = 6.049, *p* < 0.05).

### Grade at first consumption

Among drinkers, a greater proportion of boys than girls began drinking in elementary school (Table 2). Immigrants were more likely to have started drinking in elementary school (60.9 %) compared to junior or high school (39.1 %). Among Israeli-born participants, 35.1 % started drinking in elementary school compared to 64.9 % in junior or high school. Of those who first drank in elementary school, 54.5 % currently drink, compared to 34 % of those who started in junior high school and 20.8 % of those who started in high school.

**Table 2:** Characteristics of students according to school level at first consumption of energy drinks

Characteristic	Total	First consumption	
		Elementary	Junior high/High school
Gender, N (%)			
Male	352	152 (43.2)	200 (56.8)
Female	316	94 (29.7)	222 (70.3)
*χ* ^2^ = 12.919^**^			
Current school level, N (%)			
Junior high	296	143 (48.3)	153 (51.7)
High school	363	99 (27.3)	264 (72.7)
*χ* ^2^ = 31.06^**^			
Country of birth, N (%)			
Israel	612	215 (35.1)	397 (64.9)
Other	46	28 (60.9)	18 (39.1)
*χ* ^2^ = 12.17^**^			
Family structure, N (%)			
Parents married	505	172 (34.1)	333 (65.9)
Other	141	60 (42.6)	81 (57.4)
*χ* ^2^ = 3.46			
Parental education level, N (%)			
Low	193	72 (37.3)	121 (62.7)
High	397	128 (32.2)	269 (67.8)
*χ* ^2^ = 1.49			
Consumes energy drinks daily, N (%)			
No	390	110 (28.2)	280 (71.8)
Yes	261	132 (50.6)	129 (49.4)
*χ* ^2^ = 33.50^**^			
Mix energy drinks and alcohol, N (%)			
No	356	115 (32.3)	241 (67.7)
Yes	299	126 (42.1)	173 (57.9)
*χ* ^2^ = 6.76^*^			

^*^Significant *p* < 0.01; ^**^Significant *p* < 0.001

### Reasons for drinking energy drinks

When queried about reasons for drinking, the most frequent responses were 50.2 % drank because of the taste, 12.7 % drank to feel energized, 19.3 % in order to mix with alcohol, 11 % to stay awake and 5.3 % reported drinking out of curiosity. Multiple responses were allowed.

### Alcohol Mixed with Energy Drinks (AmED)

Sixty-one percent of respondents had never consumed AmED and 41.8 % of those who drank ED had never mixed them with alcohol; 13.4 % reported that they only drank ED when mixed with alcohol. In total, 47.0 % of the high school students and 30.1 % of junior high students had ever consumed AmED. Of those who drank AmED, 80.6 % drank to improve the taste of their alcoholic beverage, 24.6 % to feel intoxicated, 14.6 % out of curiosity, and 13.9 % to feel awake; whereas 11.7 % mixed to consume more alcohol, 10.4 % for social reasons, and 8.4 % to reduce side effects of the alcohol. Of those who currently were consuming AmED, 32.3 % first began drinking in elementary school. Those who started drinking in elementary school were more likely to drink currently and to consume AmED. Of those who first drank in elementary school, 52.3 % currently drink AmED, along with 41.8 % of those who started drinking ED in junior high or high school.

### Knowledge regarding energy drinks and caffeine

Many participants were aware of the likelihood of increases in heart rate and blood pressure, although that knowledge was not associated with ED use. A large percentage, (80.2 %) knew that caffeine from other sources has a cumulative effect. Most (94.1 %) knew that coffee and cola drinks (81.9 %) have caffeine, although less than half (42.8 %) knew that tea and dark chocolate (40.1 %) contain caffeine. A similar percentage (42.6 %) underestimated the amount of caffeine in a single serving can and more than 90 % thought that a can exceeded the recommended amount of caffeine. Those who believed that a can exceeded the recommended caffeine limit were less likely to consume ED (OR 1.93, 95 % CI 1.18–3.14). Slightly over half (54.1 %) had received some formal education about ED and 50.1 % said they would be interested in learning more.

### Knowledge about AmED

More than half (55.7 %) knew that ED mask the effect of alcohol. Most (71.3 %) knew that those who drink AmED were more likely to drink more alcohol than those who do not mix it with ED. Similarly, 55.2 % had learned about the risks of AmED and 51.5 % responded that they would be interested in learning more.

### Predicting Energy Drink and AmED Use

In a second level of analyses, regression equations demonstrated the relative relationship between the independent variables, gender, family structure, current school level, parental education and country of birth. In the equation with ED use as the dependent variable and gender, family structure, parental education, country of birth and school level at first consumption as independent variables, current school level, gender and school level at initial consumption were significant explanatory variables (Table 3). Other sociodemographic variables were not significant when included in the logistic regression. When AmED was the dependent variable, the same variables: current age, gender and age at first consumption were the only significant independent variables (Table 3).

**Table 3:** Logistic regressions predicting energy drink and AmED consumption

Variable	Energy drink consumption (*N* = 657)			Alcohol and mixed energy drinks (*N* = 662)		
	OR	95 % CI	P	OR	95 % CI	P
Gender (0 = Female; 1 = Male)	2.26	1.60–3.18	0.000	2.58	1.86–3.57	0.000
Family structure	1.42	0.91–2.21	0.119	1.40	0.91–2.15	0.123
(0 = Parents married; 1 = Other)						
Parental level of education (0 = High; 1 = Low)	1.25	0.87–1.79	0.230	1.35	0.96–1.90	0.089
Current school level (0 = High school; 1 = Junior high)	1.81	1.27–2.56	0.001	0.52	0.38–0.73	0.000
Country of birth (0 = Israel; 1 = Other)	2.06	1.02–4.13	0.043	0.79	0.38–1.64	0.527

We were particularly interested in the differences between early drinkers compared to those who started in junior or senior high school (Table 4). We found that for those who started drinking in elementary school, gender was the only significant predictor variable. However, for those who started drinking in junior or senior high school, gender was not significant, but current school level; having a single parent and having both parents with no more than a high school education were found significant. We found a similar pattern when AmED consumption was the dependent variable (Table 5). For those who started drinking in elementary school, gender and current school level were significant. For those who started drinking ED in junior or senior high, gender was not significant, but current school level, family structure and parental education level were all significant variables, with current school level the most highly significant and with the lowest odds ratio (0.44–0.45) (Table 5).

**Table 4:** Logistic regressions predicting energy drink consumption by school level at first consumption

	First consumption					
	Elementary School (*N* = 193)			Junior high/High school (*N* = 366)		
	OR	95 % CI	P	OR	95 % CI	P
Gender (0 = Female; 1 = Male)	2.39	1.28–4.43	.006	1.41	0.88–2.25	.149
Family structure (0 = Parents married; 1 = Other)	0.78	0.37–1.63	.503	1.82	1.01–3.28	.045
Parental education level (0 = High; 1 = Low)	0.83	0.44–1.56	.563	1.64	1.01–2.65	.046
Current school level (0 = High school; 1 = Junior high)	1.64	0.90–3.01	.107	1.73	1.08–2.79	.024
Country of birth (0 = Israel; 1 = Other)	2.85	0.91–8.91	.071	1.18	0.38–3.66	.773

**Table 5:** Logistic regression predicting energy drink and alcohol mixing by school level at first consumption

	First consumption					
	Elementary School (*N* = 192)			Junior high/High school (*N* = 372)		
	OR	95 % CI	p	OR	95 % CI	p
Gender (0 = Female; 1 = Male)	4.13	2.18–7.84	.000	1.39	0.90–2.15	.133
Family structure (0 = Parents married; 1 = Other)	0.68	0.31–1.47	.326	1.76	1.01–3.08	.047
Parental level of education (0 = High; 1 = Low)	0.88	0.45–1.72	.712	1.62	1.03–2.55	.038
Current school level (0 = High school; 1 = Junior high)	0.44	0.23–0.84	.013	0.45	0.28–0.71	.001
Country of birth (0 = Israel; 1 = Other)	1.40	0.46–4.29	.553	0.36	0.11–1.21	.100

## Discussion

This study provides important new information regarding ED use in a sample of youth 14 to 18 years of age. This sample is younger than that in the preponderance of literature. The majority (84.2 %) had ingested ED, with more than a third of those who ingested ED, (36.8 %) first beginning to drink while in elementary school and 33.8 % drinking daily. These data are comparable to that of previous reports [4] and extend those findings. For example, Simon and Mosher [3] noted that 31 % of the 12- to 17-year-olds in their study were drinking regularly. Similar to results found in Ontario Canada [19] where 50 % of students consume energy drinks and 20 % drank in the last week, our results underscore that the majority of youth have consumed energy drinks at very young ages and that early drinking is associated with increased ED and AmED consumption in high school.

Energy drink manufacturers have successfully advertised to youth [20]. Some of these companies focus on youth-oriented social media advertising and sponsor events and athletes that appeal to high school-age students. Moreover, children under 12 in the United States saw an average of 62 ED and “shot” ads in 2010, which is comparable to the number of ads that they saw for two popular children’s drinks, Capri Sun and Kool-Aid [20]. Likewise, our data indicate that young children are consuming ED. First ED consumption in our study was at a mean age of 12.5 years. Many had their first ED at home, sometimes as young as age 3.5 years. It is highly likely that parents were aware that their children were consuming ED, which suggests that educational efforts need to be targeted toward parents, as well as youth. Those who started drinking in junior or senior high school, were more likely to come from single parent homes and families with both parents with no more than high school level of education than those who began drinking earlier. This information can also help target appropriate audiences for education efforts. Those efforts include clear composition and labeling requirements and educational initiatives within schools**,** particularly in junior high.

The fact that 4.2 % of the sample reported requiring medical attention after drinking highlights the potential harm of allowing youth access to ED and the importance of increased public awareness of the risks associated with their consumption. Of note, more junior than senior high students were imbibing more than one can per day, which indicates the appeal to younger students and the importance of educating parents and students of the risks and limiting access to ED by youth.

The logistic regression analyses in this study demonstrate the strong association between early and current consumption of ED. Those who started drinking in elementary school were more than twice as likely to drink currently as those who started later. Gender and age were also predictive of current ED consumption; those who were older or male were more likely to consume ED. Those who started drinking in elementary school were twice as likely to be currently drinking.

We also found that in addition to being at greater risk for ED consumption, the risk of AmED consumption was higher amongst those who started drinking early. Most youth who start drinking at a young age, continue to drink (54.5 %). Also concerning, 52.3 % of those who started drinking in elementary school were drinking AmED at the time of the study, as well as 39.6 % of those who started in junior high. Other researchers found increasing consumption of AmED among youth populations [21, 22].

Frequent ED consumption may identify students at risk of substance use [23]. Likewise, Bernstein et al. [24] found an association between caffeine consumption and tobacco and alcohol use a year later. Arria et al. [10] found that college students who consume ED frequently (52 or more times within a year) were at a significantly higher risk for alcohol dependence and episodes of heavy drinking.

In our study, the characteristics of the early drinking group differed from those who started drinking in junior or senior high school. The most striking feature was that boys were much more likely to start drinking at a younger age. We also found that there were more immigrants among those who started drinking in elementary school, (*χ*^2^(1) = 12.169, *p* < 0.001). The risks of alcohol use and especially AmED drink use have been well described [22, 25–29].

The association between drinking ED at a young age and current AmED consumption is very important. The characteristics of early drinkers can help raise awareness regarding potential at-risk groups. For example, youth in junior or senior high school with less educated parents or single parents may be at greater risk. We also know that boys are more likely to drink ED.

It seems particularly important to limit ED in young children in order to reduce the risk of later AmED use. We cannot know whether early ED consumption makes one vulnerable to later use, or if early ED consumption and later AmED consumption occur because both are used by sensation-seeking individuals. Some research supports the latter [30, 31], whereas others have not found support [32, 33]. In either case, early identification and intervention may lead to decreased consumption of both ED and AmED. This has been found to be the case for alcohol consumption [34–36]. Although intervention programs do exist [37], no studies of actual efficacy of interventions regarding energy drink use were found.

Our data provide mixed evidence regarding the role of preventive education and knowledge. Although about half of the students had received education about ED, it did not correlate with ED use. Knowledge of health risks such as increased heart rate and high blood pressure were also not related to use. We did find that those who believed that the amount of caffeine in a can was equal or greater than the acceptable amount were less likely to ingest ED. Those who believed that there was less caffeine in a can than what they thought was the acceptable limit were more likely to drink (OR 1.93, 95 % CI 1.18–3.14). In other words, it may be that those who thought that a can contained the maximum acceptable amount were likely to ration themselves. This has clear implications for educational efforts. We also suggest that warning labels with recommended limits of caffeine consumption for youth and total caffeine amount per can might affect the decision of some young people to drink. Many studies from tobacco packaging have shown that warning labels can be effective [38–42], whereas other studies have found warnings on tobacco and alcohol to be ineffective [43, 44].

### Limitations

The study used cross-sectional data which limits the ability to assess causal relationships. Data were self-reported and students may have over- or underestimated their own consumption. In addition, those who were currently consuming ED might be more likely to remember drinking when younger; indicating potential recall bias. Lastly, the sample included students from one city in Israel. Larger studies with more diverse sampling and larger sample sizes are necessary before the results can be generalized. Although the study sample did reflect previous research findings of others, Tel Aviv does not represent the entire country and represents of a sample that is urban, Jewish and largely secular.

## Conclusions

This study demonstrates the popularity of ED among youth. More high school students drank than junior high students, but a substantial number started consuming ED as early as elementary school. We also saw that those students who started drinking in elementary school were more likely to be currently ingesting ED and AmED. We suggest that both parents and students need to be educated about the risks of ED consumption and that access to ED should be restricted. In addition, our data indicate that more information about acceptable levels of caffeine consumption and actual amounts of caffeine in a single product might lead to decreased consumption.

## Abbreviations

AmED: Alcohol mixed with energy drinks
ED: Energy drinks
